# Promoting alignment with Mediterranean and planetary health diet principles among international students: an exploratory pilot study of the MeditEat project

**DOI:** 10.3389/fnut.2026.1946581

**Published:** 2026-10-21

**Authors:** Lenycia de Cassya Lopes Neri, Kalpana Shivanandappa, Simona Fiorini, Dirce Marchioni, Mariangela Rondanelli, Cinzia Ferraris, Daniela Martini

**Affiliations:** 1 Laboratory of Food Education and Sport Nutrition, Department of Public Health, Experimental and Forensic Medicine, University of Pavia, Pavia, Italy; 2 Department of Public Health, Experimental and Forensic Medicine, Human Nutrition and Eating Disorder Research Center, University of Pavia, Pavia, Italy; 3 Department of Earth and Environmental Sciences, University of Pavia, Pavia, Italy; 4 Department of Nutrition School of Public Health, University of São Paulo, São Paulo, Brazil; 5 Department of Food, Environmental and Nutritional Sciences (DeFENS), Università degli Studi di Milano, Milan, Italy

**Keywords:** diet acculturation, international students, Mediterranean diet, PHDI, sustainability

## Abstract

**Introduction:**

The global food system is a major driving force behind environmental degradation, and dietary shifts towards more resource-efficient plant-based patterns are critical for reducing ecological pressure. The Mediterranean diet is widely recognized for its high nutritional quality and low environmental footprint, making it a relevant model for promoting sustainable eating behaviours also among mobile populations such as international students. This study evaluates dietary sustainability defined by the Planetary Health Diet Index (PHDI) among international students at baseline and the changes observed during the effect of a structured nutrition education exploratory pilot program (MeditEat).

**Methods:**

Fifteen students from diverse cultural backgrounds participated in MeditEat. The program included three in-person meetings and three online meetings, with monthly assessments based on the Mediterranean diet Pyramid and practical nutritional counseling to support the application of these concepts. Participants completed food-frequency questionnaires at baseline (T0), 3 months (T3), and 6 months (T6). Dietary data were converted into daily caloric contributions per food group using standardized procedures. The modified Planetary Health Diet Index (PHDI) was calculated, with a maximum possible score of 140. Statistical analyses, including the Shapiro–Wilk test, repeated-measures ANOVA, and Friedman test, were performed to evaluate time-related variations.

**Results:**

Total PHDI score increased significantly from baseline (75.4 ± 13.8) to T6 (82.9 ± 13.6, *p* = 0.01). Vegetable oil was the only individual component to show a statistically significant increase (*p* = 0.004), alongside non-significant descriptive trends in other components.

**Conclusion:**

Although variability across individuals remained considerable, participation in the pilot programme was associated with preliminary trends toward improved alignment with sustainable dietary patterns. The observed modest increase in PHDI score may indicate a favourable trend associated with Mediterranean diet-based nutritional education, although further research in larger, well-designed controlled studies is needed to substantiate this finding.

## 1 Introduction

In recent years, a growing interest has arisen regarding the multitude of global diets and their effects on population health and growth, as well as their environmental and economic impact (1). This intrinsic relationship between nutrition and the environment is well established, as eating patterns affect the environment and vice versa (2–5). Technological advances, globalization and changes in agricultural systems have caused changes in the human diet and the current global food system is increasingly associated with human health and well-being, as food insecurity and malnutrition in all its forms, as well as significant environmental damage (6–8). The need for sustainable diets has been recognized, and they should be culturally acceptable, economically accessible, safe and healthy (4, 5). It is important to respect these principles in order to minimize negative impacts on biodiversity and ecosystems and optimize both natural and human resources (9–11).

In this context, the Mediterranean diet is widely recognised as a traditional eating pattern rooted in the food cultures of Mediterranean countries (1, 12, 13). Rather than representing a single fixed model, it reflects a variety of dietary patterns that share a common set of core characteristics: large consumption of plant-based foods, such as whole grains, fruits, vegetables and legumes and regular use of extravirgin olive oil. These foods are complemented by moderate consumption of fish, dairy products, white meats and eggs, while red and processed meats remain limited, supporting both nutritional adequacy and environmental balance (14). Beyond its nutrient profile, the Mediterranean diet incorporates important values such as seasonality, biodiversity, and the preference for locally sourced foods, contributing to its positioning as a sustainable and culturally meaningful dietary pattern (15, 16). Extensive evidence links this diet to favourable metabolic and cardiovascular outcomes, further reinforcing its relevance in modern nutrition (17, 18).

Introducing international students to the key principles of the Mediterranean diet may help them make healthier choices and maintain sustainable eating habits over the long term (19). The growing internationalization of higher education has led to a significant rise in student mobility across countries. International students, who temporarily reside abroad for academic purposes, contribute to cultural exchange and global collaboration after graduation (20, 21). Living in a new cultural environment may involve a process of acculturation, whereby individuals partially adapt to local customs maintaining their original cultural identity. This transition often brings linguistic, social, and financial challenges, influencing lifestyle and food choices. Dietary acculturation, as part of this process, requires adjustment of eating habits, ingredients, and meal patterns of the new environment, which may have both positive and negative effects on health (22, 23). Recent qualitative evidence among international university students highlights that dietary behaviors and choices during cultural adaptation are shaped not only by nutritional knowledge, but also by broader financial, psychosocial, and academic pressures (24). For instance, among French university students, higher cooking skills were associated with improved nutritional quality through greater intake of vegetables, legumes, and grains (25). On the other hand, the shift toward Western dietary patterns, often characterised by fat-rich and high-sugar food consumption, as frequently observed in the United States, may elevate the risk of developing chronic diseases (22, 23, 26).

Based on these premises, the present study aims to: (I) assess alignment with Planetary Health Diet principles (using the PHDI) among international students upon arrival in Italy and (II) to evaluate dietary changes associated with participation in a structured nutrition education exploratory pilot program, the MeditEat project.

## 2 Materials and methods

This is a non-randomized, uncontrolled, one-arm pilot intervention study. A more thorough description of the methodological aspects of the MeditEat project has already been published and covered in a recent publication (27). The protocol was approved by the Lombardy Territorial Ethical Committee on April 09th 2024 – number: 0024013/24. Enrolled participants consented to the anonymous use of personal data for the study. All enrolled participants provided written informed consent prior to inclusion in the study, including consent for the anonymous use of their personal data.

### 2.1 Study population

The sample was recruited among international students enrolled at the University of Pavia, Italy, through the Internationalization Office, residential colleges, flyers, and notice boards. Participation was voluntary, and students decided spontaneously to join the project. Inclusion criteria were a minimum age of 18 years old, non-Italian nationality, current enrolment at the University of Pavia, Italy and ability to understand English or Italian. Exclusion criteria included pregnancy, diagnosed mental health conditions or associated therapeutics, medications affecting appetite or dietary intake, and self-reported medical conditions that impaired the ability to follow dietary guidance. Food allergies were not an exclusion criterion.

### 2.2 Baseline psychological and lifestyle determinants assessment

At baseline (T0), participants completed a structured non-validated lifestyle questionnaire evaluating psychological and lifestyle determinants on a 0-to10 scale (where 0 indicated the lowest and 10 the highest score). Specifically, the assessment included self-reported stress, homesickness, familiarity with dietary guidelines (from both their origin country and Italy), emotional well-being and relationship with food, coping mechanisms, and social support related to dietary changes. Qualitative open-ended questions captured self-reported dietary changes since arriving in Italy and the perceived primary drivers behind those changes (such as time availability, economic constraints, and local food access).

### 2.3 Intervention design

The study involved a structured six-session nutritional education program, delivered on a 1:1 (individualized) basis over a total duration of 6 months, alternating three in-person meetings (held at baseline/session 1 [T0], session 3 [T3], and session 6 [T6]) with three online follow-up sessions (held after 1 month – session 2 [T2], session 4 [T4], and session 5 [T5]). To accommodate students’ academic calendars and personal availability, each session was delivered individually with flexible, personalized scheduling. All sessions were delivered in English to support international participants. Full dietary and anthropometric assessments were performed at three primary evaluation time points: baseline (T0), 3 months (T3), and 6 months (T6). Participants were requested to complete all assessment questionnaires prior to attending each in-person meeting. In a few logistical instances, participants handed in completed questionnaires immediately following the educational session. Crucially, because the Food Frequency Questionnaire (FFQ) measures habitual dietary intake over the preceding monthly period, completing it immediately post-session reflects prior dietary habits and does not allow time for immediate post-session behavioral modification. Before each in-person visit, students completed these validated questionnaires:

- An English-translated Food frequency questionnaire (FFQ) containing 110 items (28) was administered at each dietary assessment time point (T0, T3 and T6). This FFQ was previously tested for reliability and relative validity among individuals living in Sicily, southern Italy. Due to the difficulty of using a single cross-culturally validated questionnaire applicable to all participant origins, a FFQ tailored specifically to typical Italian foods was employed as participants were residing in Italy. To account for cultural foods, complex recipes were decomposed into base ingredients and mapped to the closest equivalent FFQ items, with nutrient and energy intakes derived using the Italian Food Composition Database (BDA). Standard portion sizes were defined in grams. This FFQ was used for a posteriori calculations of the Planetary Health Diet Index (PHDI).
- Mediterranean Diet Score (MEDI-LITE) (29) is a validated tool with 9 items, which takes an average of 3–5 min to complete, for measuring adherence to the Mediterranean diet. It assigns points based on the frequency of consumption of nine food categories: (1) fruit; (2) vegetables; (3) cereal grains; (4) legumes; (5) fish and fish products; (6) meat and meat products; (7) dairy products; (8) alcohol intake and (9) olive oil. The final score is obtained by summing these values, and it varies from 0 (lowest adherence) to 18 (highest adherence).

Every in-person session incorporated body weight measurements and body composition evaluation through skinfold thickness and bioelectrical impedance analysis, as detailed in the original protocol (27).

The intervention consisted of six structured monthly sessions combining theoretical nutrition education with practical, actionable guidance scheduled across the six-month intervention period (Figure 1). To define the exact quantity, sequence, and modality of the intervention:

- Session 1 (T0 - In-Person Baseline Meeting): Introduced the updated Mediterranean diet pyramid as defined by the Italian Society of Human Nutrition (SINU) (1), emphasizing a plant-based foundation rich in fruits, vegetables, and whole grains, with extra-virgin olive oil as the primary fat source, alongside moderate intake of dairy, eggs, and fish, and limited consumption of red meat and sweets.
- Session 2 (Online Follow-up): Focused on daily and weekly tiers for legumes, nuts, spices, and dairy.
- Session 3 (T3 - In-Person Meeting & Intermediate Dietary Assessment): Addressed animal-protein sources and sweets, reinforcing moderation.
- Session 4 (Online Follow-up): Covered practical skills including food label reading, hydration, and the social aspects of conviviality.
- Session 5 (Online Follow-up): Emphasized physical activity as an integral lifestyle component.
- Session 6 (T6 - Final In-Person Meeting & Assessment): Concluded the program with post-intervention assessments (measurements and questionnaires) and a practical session on sustainability, biodiversity, and seasonality (1, 27).

**Figure 1:**
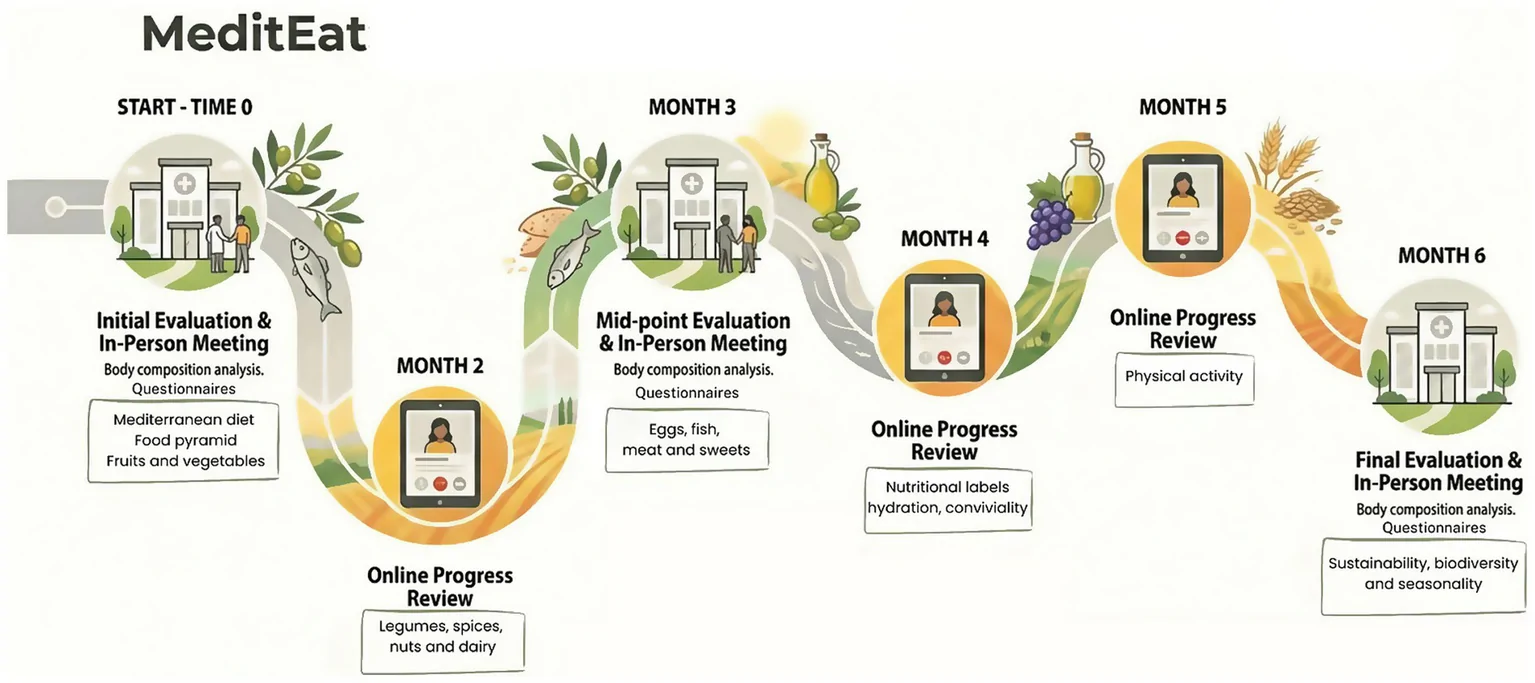
Schematic timeline of MeditEat with topics and modality of food education sessions.

To ensure optimal clarity and facilitate the assimilation of key dietary guidelines, each session integrated comprehensive visual aids and graphical materials.

### 2.4 Intake calculations and data processing

The food items included in the FFQ were grouped according to their nutrient content into eight food groups: meat and fish products; sweets, nuts, and snacks; vegetables; fruits; beverages; cereals and starchy foods; milk and dairy products; and oils and seasonings. FFQ frequency categories were standardized into estimated daily intakes (g/day and corresponding kcal per food item). When portion sizes were not explicitly reported, standardized average portion weights were derived from the *Consiglio per la ricerca in agricoltura e l’analisi dell’economia agraria* (CREA) (30) food composition database.

Complex foods or mixed dishes (e.g., pizza) were disaggregated into their constituent ingredients using standard recipe composition ratios, and their caloric contributions were allocated to the appropriate food groups. Total daily energy intake was calculated as the sum of caloric contributions from all food items, and the relative contribution of each food group was expressed as a percentage of total daily caloric intake. An Excel spreadsheet was used to automate these conversions and link each food item to the corresponding PHDI component to streamline the scoring process and reduce approximations and the chance of calculation errors.

### 2.5 PHDI scoring

Among the various indices available in the literature for assessing dietary pattern sustainability, the PHDI was selected for its ability to normalize data according to total energy intake (31). The modified PHDI index (32) comprises 15 components distributed across adequacy, optimum, moderation, and ratio domains. For each participant at T0, T3 and T6, the percentage of daily calories from each food group was computed and scored according to published thresholds (32). Adequacy components were assigned maximum points once minimum thresholds were reached; moderation components decreased from 10 to 0 points depending on excessive intake; optimum components used linear interpolation to reflect deviations between the recommended levels and the maximum allowed; ratio components evaluated proportional contributions of dark green and red/orange vegetables to total vegetable intake. Intermediate scores for all components were calculated using linear interpolation between defined cutoff values (exact formulas are provided in Supplementary Table S1). The total PHDI score ranged from 0 to 140: 10 points maximum for 13 of the components and 5 for the red vegetable (ReV) and dark green vegetable (DGV) groups. Due to the difficulty of accurately isolating animal fat intake from animal-based foods assessed by the FFQ, the animal fat component was excluded from the calculation, reducing the total maximum index from 150 to 140 points. To avoid confusion between raw food intake and PHDI component scores, higher scores in adequacy and optimum components reflect greater dietary intake toward target thresholds, whereas higher scores in moderation components reflect reduced intake of restricted foods.

### 2.6 Statistical analysis

The original sample size calculation was aimed at detecting changes in Mediterranean diet adherence (27). Given the absence of prior studies on this subject, a large effect size of *f* = 0.5 (33), an alpha error of 1%, and a power of 90% were assumed, yielding a required sample size of 14 students (34). The procedure was conducted using the calculation guidelines described by Cohen (35) and operationalized according to the method implemented by Cohen (35). Importantly, no *a priori* power analysis was performed for the PHDI outcome, and the sample size calculation was not powered for this index. Thus, all analyzes with the PHDI should be considered purely exploratory and hypothesis-generating. However, with only 15 participants, we cannot determine statistical power, particularly given the small number of subjects relative to the large number of individual PHDI components. Data distribution for each PHDI component at each time point was assessed using the Shapiro–Wilk test. Normally distributed variables were analysed using repeated-measures ANOVA; non-normally distributed variables were analysed using the Friedman test. When the tests were significant, *post hoc* comparisons with appropriate corrections were applied. Statistical analyses were conducted using JASP software (36), and a significance threshold of *p* < 0.05 was adopted.

## 3 Results

A total of 17 students were screened, met inclusion criteria, and were enrolled. During the 6-month period, 2 participants discontinued (one due to academic time constraints and one due to depression, an exclusion criterion). A total of 15 participants completed all assessments (T0, T3, T6) and were included in the final complete-case analysis (Supplementary Figure S1), yielding an overall program retention rate of 88.2% (15/17). Participant attendance across all six individual sessions reached 100% (15/15, 100%) for completing participants, supported by the 1:1 format and flexible personalized scheduling. Participant engagement during both in-person and online sessions was consistently high across the 6-month intervention. In these individual consultations, students actively engaged in tailored discussions to adapt their traditional dietary practices to Mediterranean diet principles. Qualitative observations highlighted strong participant interest in learning how to identify local Italian food ingredients, reconstruct traditional home recipes using sustainable regional alternatives, and incorporate seasonal produce into daily meal planning. This direct interaction demonstrated high intervention acceptability, facilitating individualized dietary acculturation. The age distribution clustered between the mid-20s and early 30s, in line with their role as students of various levels (undergraduate level, master’s or doctoral), showing an average age of approximately 30.1 ± 5.9 years (ranging from 21 to 43). In terms of academic level, 40.0% (*n* = 6) were Master’s students, 26.7% (*n* = 4) were undergraduates, 20.0% (*n* = 3) were PhD candidates, and 13.3% (*n* = 2) were in post-graduate specialization. The participants represented a set of different nationalities, including India, Iran, Guatemala, Turkey, Jordan, Germany, Peru, Brazil, Mexico, Myanmar, and Portugal, representing a culturally varied group (Figure 2). At the same time, the majority of participants were female (73.3%, *n* = 11), with 26.7% (*n* = 4) male participants. Regarding living arrangements, 46.7% (*n* = 7) resided in university college dormitories, 26.7% (*n* = 4) shared apartments with peers, and 20.0% (*n* = 3) lived with a partner or family. Primary cooking facilities and practices included cooking at home (46.7%, *n* = 7), utilizing university canteens (13.3%, *n* = 2), or not cooking in Italy (20.0%, *n* = 3). A history of previous weight-loss diets was reported by 60.0% (*n* = 9) of participants, and 33.3% (*n* = 5) noted specific food allergies or intolerances (e.g., lactose, nickel, or food-related sensitivities). Participants arrived in Italy between 2022 and early 2025, resulting in varying durations of residence before baseline assessment (ranging from under 1 month up to 2 years).

**Figure 2:**
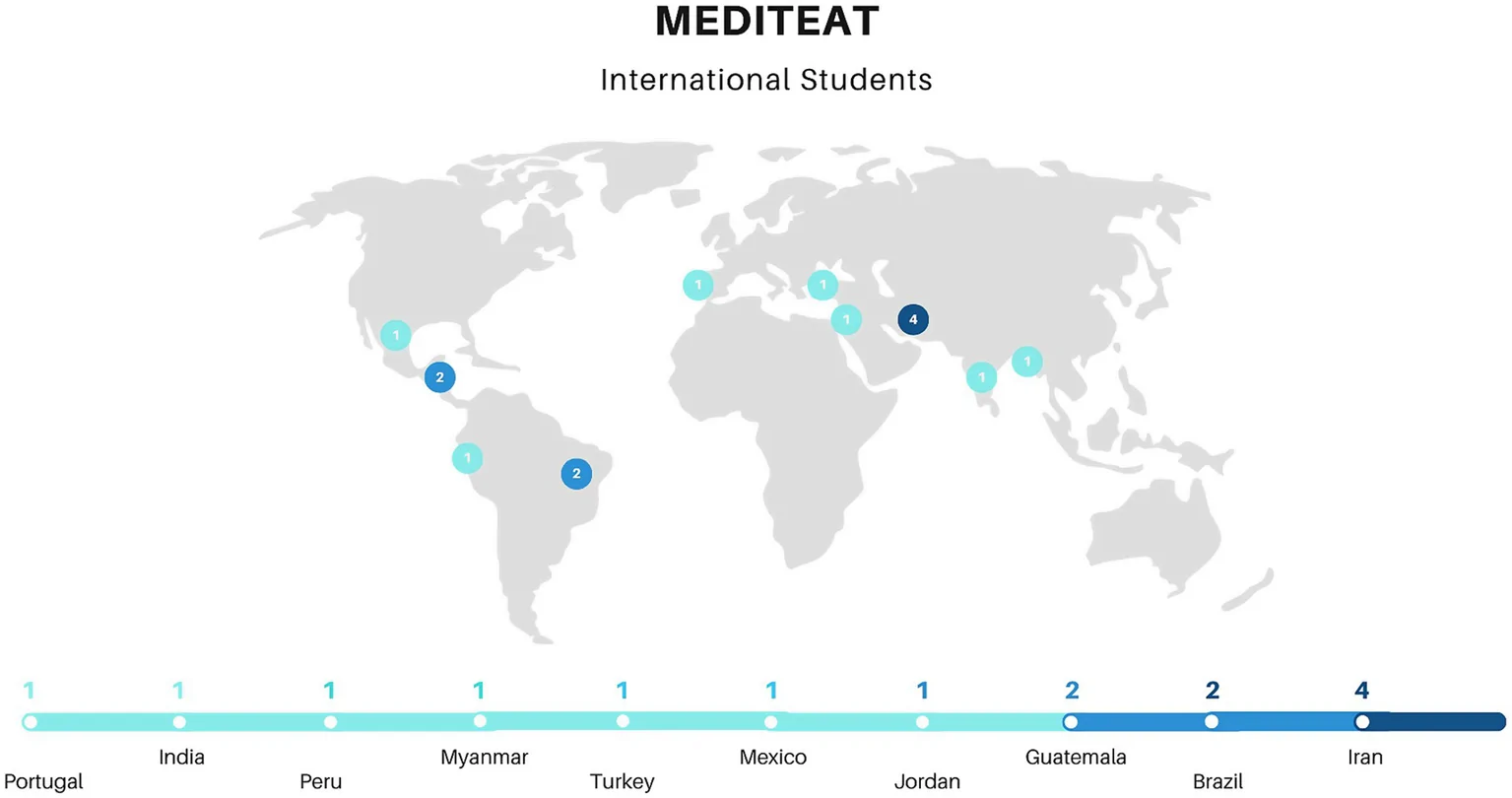
Distribution of nationalities from the sample.

At baseline (*n* = 15), self-reported ratings revealed low familiarity with Italian nutritional guidelines compared to guidelines from participants’ origin countries, alongside moderate levels of stress and homesickness (Table 1). Qualitative responses regarding dietary shifts upon arrival in Italy revealed that the primary reported causes for dietary changes were time constraints and academic schedules (26.7%, *n* = 4) and differences in local food availability or options (26.7%, *n* = 4), followed by differences in taste (13.3%, *n* = 2) and economic difficulties (6.7%, *n* = 1).

**Table 1:** Baseline characteristics of the sample.

Variables	Median	Mean	Std. deviation	Minimum	Maximum	25th percentile	75th percentile
Age (years)	32.0	30.9	6.2	20.0	42.0	27.0	34.5
BMI (kg/m^2^)	23.8	24.9	4.9	17.6	33.5	20.9	28.2
Fat mass (%)	31.7	32.0	9.5	17.9	55.4	24.7	37.7
MEDI-LITE (score)	7.0	6.5	1.0	5.0	8.0	6.0	7.0
Self-reported stress*	5.9	6.5	3.1	0.0	10.0	4.0	8.5
Homesickness*	4.6	5.0	2.7	1.0	9.0	2.0	7.0
Knowledge of guidelines (Origin Country)*	7.0	6.0	3.0	0.0	10.0	3.8	8.0
Knowledge of guidelines (Italy)*	2.0	2.4	2.6	0.0	7.0	0.0	4.5
Emotional well-being and relationship with food*	7.0	6.9	2.6	0.0	10.0	6.0	8.8
Coping mechanism*	7.0	6.7	1.5	4.0	9.0	5.0	8.0
Social support related to dietary changes*	7.0	5.9	4.3	0.0	10.0	1.5	10.0

BMI, Body mass index.

*Rated by the participant in a scale from 0 (lowest) to 10 (highest).

### 3.1 Total PHDI score

The mean total PHDI score increased significantly from 75.4 ± 13.8 at baseline (T0) to 82.8 ± 12.2 at T3 (post-hoc p-holm T0 vs. T3 = 0.03) and remained stable at 82.9 ± 13.6 at T6 (post-hoc p-holm T3 vs. T6 = 0.96). Post-hoc comparisons between T0 and T6 confirmed a significant difference (*p* = 0.03). This pattern suggests a hypothesis of early dietary modification following exposure to foundational Mediterranean diet principles between T0 and T3, followed by maintenance of these changes through T6. However, because dietary outcomes were measured across three primary assessment time points (T0, T3, T6) rather than monthly, these observations remain exploratory hypotheses rather than proof of specific intervention effects. Furthermore, this pilot study cannot determine the exact timing of dietary shifts or isolate the effect of individual educational modules. Distribution changes across time points (T0, T3, T6) for the Planetary Health Diet Index (PHDI) total score and its individual components are illustrated using raincloud plots (displaying individual data points, medians with interquartile ranges, and density distributions) in Supplementary Figures S2–S15.

### 3.2 Adequacy components

Mean scores for nuts and peanuts increased progressively from T0 (4.1 ± 3.4) to T6 (5.1 ± 3.6), though changes were not statistically significant (*p* = 0.57). A similar trend of improvement was observed for legumes, although changes were not statistically significant. Fruits and vegetables intake remained high, consistent with Mediterranean dietary recommendations. Whole cereals displayed a non-significant trend of reduction [median 8.4 (6.5–10) at T0, median 6.0 (4.9–8.0) at T6; *p* = 0.13], possibly reflecting substitution with other plant-based foods introduced during counseling (see Table 2).

**Table 2:** Summary table of sustainability scores for different food groups, according to the Mediterranean diet intervention timing.

Variable	T0	T3	T6	*p*-value	Effect Size
	Score	Score	Score		
Nuts	4.1 ± 3.4	4.9 ± 3.5	5.1 ± 3.6	0.57^a^	0.017
Legumes	3.1 (1.5–5.4)	4.0 (2.5–8.4)	5.0 (3.3–7.6)	0.74^b^	0.020
Fruits	10.0 (8.8–10.0)	10.0 (7.0–10.0)	10.0 (8.8–10.0)	0.20^b^	0.106
Vegetables	10.0 (5.8–10.0)	10.0 (7.8–10.0)	10.0 (9.4–10.0)	0.61^b^	0.033
Cereals	8.4 (6.5–10.0)	8.1 (2.8–9.3)	6.0 (4.9–8.0)	0.13^b^	0.138
Eggs	0.0 (0.0–3.1)	0.0 (0.0–2.5)	0.0 (0.0–4.0)	0.68^b^	0.025
Fish and Seafood	3.5 ± 3.2	3.8 ± 3.1	3.6 ± 3.6	0.97^a^	0.001
Tubers	4.9 ± 2.9	5.5 ± 2.6	4.4 ± 3.4	0.51^a^	0.024
Dairy	0.0 (0.0–4.3)	0.0 (0.0–5.2)	0.0 (0.0–5.0)	0.97^a^	0.002
Vegetables Oils	2.1 ± 1.1^c^	4.3 ± 2.8^d^	3.7 ± 2.3^d^	0.004^a^	0.160
DGV/Tot	3.6 (1.6–4.0)	3.5 (1.9–3.8)	2.4 (1.8–2.8)	0.77^b^	0.018
ReV/Tot	3.2 ± 1.0	3.4 ± 0.9	3.5 ± 1.2	0.83^a^	0.011
Red Meat	0.0 (0.0–3.9)	4.0 (0.0–6.3)	2.5 (0.0–4.7)	0.18^b^	0.115
Chicken and Subs	1.7 (0.4–4.7)	5.3 (0.3–8.9)	2.3 (0.1–8.2)	0.23^b^	0.097
Added Sugar	7.0 (5.9–9.7)	8.6 (7.3–9.5)	8.9 (7.0–9.8)	0.24^b^	0.096
Total Score	75.4 ± 13.8^c^	82.8 ± 12.2^d^	82.9 ± 13.6^d^	0.01^a^	0.071

SD, standard deviation; DGV/Tot, Dark Green Vegetables to Total vegetables calories ratio; ReV/Tot, Red and Orange Vegetables to Total vegetables calories ratio; Mean score is shown with ±SD for normally distributed data and Median (p25–p75) for non-normally distributed data; T0: Baseline; T3: 3 months of intervention; T6: 6 months of intervention; ^a^ANOVA (effect size measured by η^2^G); ^b^Friedman (effect size measured by Kendall’s W); ^c,d^Values within a row sharing different superscript letters indicate statistically significant pairwise differences across time points (*p* < 0.05, Holm *post-hoc* test).

### 3.3 Optimum components

Non-significant changes were observed in the food groups eggs, dairy products, and fish, which remained below optimal thresholds throughout the study, despite a trend of improvements being noted at T6. Vegetable oils showed the most substantial change, with statistically significant results. The PHDI component score for vegetable oils doubled from T0 (2.1 ± 1.1) to T3 (4.3 ± 2.8), partially stabilizing thereafter (3.7 ± 2.3, *p* = 0.004). Post-hoc analysis confirmed the significant difference between T0–T3 (pholm = 0.011) and T0–T6 (pholm = 0.011), but no difference was observed between T3 and T6 (pholm = 0.426). This corresponds with targeted counseling encouraging olive oil consumption, which is one of the main characteristics of the Mediterranean diet.

### 3.4 Moderation components

Although not statistically significant, red meat intake showed a decreasing trend, which led to improved moderation component scores over time (T0: 1.7 ± 2.6; T3: 3.5 ± 3.2; T6: 2.7 ± 2.7; *p* = 0.18). Chicken and substitutes followed a similar trajectory, with reduced intake reflected by higher moderation scores in the first 3 months of intervention (T0: 1.7 (0.4–4.7); T3: 5.3 (0.3–8.9); T6: 2.3 (0.1–8.2); *p* = 0.23). Added sugars showed a decreasing trend across the intervention period, with the corresponding moderation score increasing from 7.0 (5.9–9.7) at baseline to 8.6 (7.3–9.5) in T3, and finally 8.9 (7.0–9.8) (*p* = 0.24), suggesting potential trends toward healthier choices.

### 3.5 Ratio components

The distribution between dark green vegetables and red/orange vegetables remained balanced across all time points, with only slight shifts that did not reach statistical significance.

## 4 Discussion

The findings from this study suggest that participation in the MeditEat Project was associated with preliminary improvements in dietary sustainability, with PHDI scores rising at each assessment point, despite the relatively short intervention period. The greatest increase in total PHDI score occurred between baseline (T0) and 3 months (T3), after which scores stabilized through 6 months (T6). This trajectory can be best understood by examining the intervention schedule and questionnaire protocol: participants were instructed to complete assessment questionnaires prior to each session, though in isolated instances, submissions occurred immediately post-session. Because the validated FFQ evaluates retrospective dietary intake over the preceding month, responses captured established habitual intake without opportunity for immediate session-induced recall bias. Therefore, the recorded improvement at T3 reflects the cumulative behavioral modifications adopted across the initial three-month phase. During this early period, participants received three structured educational sessions (Sessions 1–3) covering foundational Mediterranean diet principles—namely plant-based staples, extra-virgin olive oil, legumes, nuts, animal protein moderation, reduced sugar consumption, and practical food selection skills. This concentrated initial exposure provided participants with the core knowledge and practical tools necessary for prompt dietary adaptation, explaining why the main improvement occurred during the first 3 months. Subsequent sessions (Sessions 4, 5 and 6) expanded into lifestyle integration, physical activity, conviviality, and broader environmental sustainability concepts, which supported the maintenance and consolidation of these adopted dietary changes through T6. While this pattern suggests an early behavioral adaptation followed by habit stabilization, the lack of monthly outcome evaluations requires this interpretation to remain an exploratory hypothesis rather than definitive proof of any individual session’s effect. Without continuous monthly data points, this pilot study cannot determine whether the dietary shift was progressive or hit an early plateau, nor can it isolate which specific educational module was directly responsible for the observed improvements.

As an *a priori* power analysis was not conducted for the PHDI, these analyses remain exploratory and hypothesis-generating. These preliminary, hypothesis-generating observations indicate that structured and personalised nutrition education warrants further investigation as a strategy to foster healthier and more environmentally responsible eating patterns in line with EAT-Lancet and Planetary Health Diet principles. While an increased intake of vegetable oils was the only individual component change to reach statistical significance (*p* = 0.004), non-significant descriptive trends were observed toward increased alignment in legumes and nuts, alongside reduced intake of red meat and added sugars. Because these descriptive trends in individual food groups did not reach statistical significance, they cannot be directly linked to environmental or health outcomes within the scope of this study. In line with the broader conclusions of Willett et al. (3), our analysis demonstrates that activities aimed at increasing dietary and health awareness may encourage the adoption of healthier, more sustainable food choices. As observed even in our relatively small sample group, the increased vegetable oil component score, together with a trend toward higher scores for legumes and nuts, provide preliminary evidence of the implementation of Mediterranean dietary recommendations. These dietary shifts, as described by Guasch-Ferré and Willett (14), can lead to long-term healthier living and a reduction of risk factors associated with severe health conditions. However, given the small sample size (*n* = 15) and the high number of PHDI sub-components analyzed, these results should be viewed as preliminary insights that justify future research adequately powered, controlled trial, rather than definitive confirmation of the intervention’s efficacy.

At baseline, many participants displayed eating behaviours characteristic of adapting to an unfamiliar dietary environment, reflected in a low self-reported knowledge of Italian nutritional guidelines (2.4 ± 2.6 out of 10) compared to their origin country guidelines (6.0 ± 3.0 out of 10; Table 1). Qualitative feedback confirmed that these initial dietary adjustments were primarily driven by time constraints and academic schedules (26.7%) and limited access to culturally familiar food options (26.7%), with economic constraints also contributing (6.7%). These baseline observations align with broader international student literature reporting reliance on convenient options during initial cultural transitions,financial pressures, and the absence of family routines (22, 23, 37–42). Specifically, lower baseline scores for certain optimal food groups reflect practical hurdles such as food affordability, market availability, and unfamiliarity with local preparation methods (21, 22, 39, 42). Although dietary acculturation dynamics vary by origin country, the small sample size of this pilot study precluded subgroup analyses by region.

University-based research consistently shows that interventions which integrate theoretical education with practical skill-building are more effective at reducing reliance on convenience foods and fostering long-term behavioural change (19, 43, 44). In line with these findings, our results suggest that the MeditEat Project’s personalised nutrition education and hands-on approaches, such as ingredient selection guidance and step-by-step meal planning, were associated with students’ improved ability to adopt Mediterranean diet principles. The 1:1 delivery format and personalized scheduling played a pivotal role in ensuring complete session attendance (100% among completers) while allowing tailored counseling that directly addressed individual student queries regarding local ingredient identification, recipe adaptation, and produce seasonality. This is further supported by Watanabe and colleagues (45), reporting that enhanced culinary skills significantly increase adherence to Mediterranean dietary patterns, particularly in populations that initially lack familiarity with such cooking methods. By replacing simple, quick calorie sources with culturally transferable plant-based alternatives, students demonstrated not only improved nutritional quality but also a stronger engagement with sustainable dietary choices, illustrating how skill-based education may help address many of the practical barriers faced by international students.

Although cardiometabolic, inflammatory, genetic biomarkers, or environmental footprint biomarkers were not directly measured in the present study, no direct inferences regarding systemic health or ecological benefits can be drawn from our data. While external literature links increased plant-based food intake and trends toward reduced consumption of red meat and sugars and having long-term benefits on cardiometabolic health, systemic inflammation, genetic risk factors, and overall well-being (46, 47), our findings are limited to self-reported dietary intake and a moderate increase in total PHDI score. Furthermore, greater adherence to the Mediterranean diet has been linked to improved subjective well-being and reduced stress among university students (48), suggesting that the dietary shifts observed here could potentially confer similar psychosocial advantages over time. Future studies incorporating objective physiological biomarkers and lifecycle assessment metrics are required to evaluate whether the moderate dietary shifts observed here translate into measurable health or environmental benefits.

### 4.1 Process-related challenges and implementation insights

Additional lifestyle and environmental factors—such as the “healthy immigrant effect” (49), marketing influences, and institutional food settings (50, 51), —further contextualize the complex interplay of cultural, economic, and environmental factors shaping student dietary transitions. These considerations suggest that future interventions should not only provide nutrition education but also address affordability, food access, and cultural adaptability to support adherence to sustainable dietary patterns.

Implementing a lifestyle and dietary intervention within an international university student cohort involved several unique challenges. Recruitment and retention required extensive outreach due to the highly transient nature of mobile student populations. Retention was also influenced by academic schedules and individual health events (e.g., time constraints and depression leading to participant dropout). These implementation barriers align with observations by Hilal et al. (24), who emphasized that academic workload and psychosocial distress frequently interact with financial and food access constraints in mobile student populations. Adapting dietary assessments for participants from 11 different nationalities was a major operational challenge, necessitating the breakdown of complex traditional recipes into base ingredients to align with Italian food composition databases. Additionally, environmental factors such as rigid academic timetables, limited food preparation time, financial constraints, and unfamiliarity with local grocery markets continually affected participants’ daily food choices during their cultural transition (21, 22, 39, 49). Although food insecurity was not formally evaluated in the present study, these contextual insights reinforce the rationale for incorporating food affordability, structural access, and cultural adaptability alongside nutrition education in future student-focused interventions (24).

### 4.2 Limitations and strengths

Several methodological limitations and uncertainties should be considered when interpreting these preliminary outcomes. First, the non-randomized, single-arm exploratory design with a small sample size (*n* = 15), no control group limits the ability to draw definitive causal conclusions about the intervention’s isolated effects, and voluntary recruitment may have introduced self-selection bias. Second, an *a priori* power calculation was not conducted for the PHDI outcome; the sample size calculation was based entirely on Mediterranean diet adherence (27). A final sample size of 15 participants lacks adequate statistical power to draw firm conclusions across the multiple individual PHDI components, requiring these findings to be viewed strictly as preliminary and hypothesis-generating. Third, because dietary sustainability outcomes were evaluated at three discrete time points (T0, T3, T6) rather than monthly, the study cannot determine the precise timing of dietary change, assess whether trajectory fluctuations occurred, or identify which specific educational module was directly associated with the observed improvements. Fourth, reliance on self-reported food frequency questionnaires may introduce recall and social desirability biases, and the Italian-validated tool might not fully capture niche traditional foods from an international cohort despite recipe disaggregation. Finally, the Planetary Health Diet Index is a surrogate measure of dietary alignment and does not directly assess ecological footprint metrics like greenhouse gas emissions, land use, water use, or food waste (51).

Notwithstanding these limitations, key strengths include the project’s focus on immediate behavioral shifts, a longitudinal repeated-measures design, and a culturally diverse sample. Combining personalized counseling with visual dietary education provided realistic insights into real-world barriers.

### 4.3 Recommendations for future research

Building on this pilot study’s exploratory findings, several key directions are recommended for future research. Future studies should focus on multi-center randomized controlled trials with larger, adequately powered samples to compare structured nutritional guidance against standard controls. To validate self-reported dietary changes, research should incorporate objective cardiometabolic biomarkers and lifecycle assessment tools that measure environmental impacts such as carbon and water footprints. Longer post-intervention follow-ups of 12 to 24 months are needed to assess the sustainability of dietary habits over students’ academic careers. Lastly, developing and validating flexible, cross-cultural dietary assessment tools will enhance tracking accuracy in diverse university populations.

## 5 Conclusion

In conclusion, this exploratory pilot study offers preliminary, hypothesis-generating evidence that short-term, structured exploratory pilot intervention may support international students in adopting more sustainable dietary habits. The observed moderate increase in total PHDI score, driven primarily by increased vegetable oil consumption, justifies further research into combining personalized counselling with practical nutrition education without serving as confirmation of overall efficacy or environmental or health benefits. Given the small sample size and lack of an *a priori* power calculation for the PHDI, these preliminary findings need to be validated in larger, adequately powered, controlled trials.

## Data Availability

The raw data supporting the conclusions of this article will be made available by the authors, without undue reservation.

## References

[1] Sofi F Martini D Angelino D Cairella G Campanozzi A Danesi F . Mediterranean diet: why a new pyramid? An updated representation of the traditional Mediterranean diet by the Italian Society of Human Nutrition (SINU). Nutr Metab Cardiovasc Dis. (2025) 35:103919. doi: 10.1016/j.numecd.2025.103919, 40087038

[2] Wiseman SA Dötsch-Klerk M Neufingerl N Martins F d O. Future food: sustainable diets for healthy people and a healthy planet. Int. J. Nutrol. (2019) 12:23–8. doi: 10.1055/s-0039-1695714

[3] Willett W Rockström J Loken B Springmann M Lang T Vermeulen S . Food in the Anthropocene: the EAT-lancet commission on healthy diets from sustainable food systems. Lancet. (2019) 393:447–92. doi: 10.1016/S0140-6736(18)31788-4, 30660336

[4] Neta RSO Lima SCVC Nascimento LL Souza CVS Lyra CO Marchioni DML . Indices for measurement of sustainable diets: a scoping review. PLoS One. (2023) 18:e0296026. doi: 10.1371/journal.pone.0296026, 38117768 PMC10732454

[5] Rockström J Thilsted SH Willett WC Gordon LJ Herrero M Hicks CC . The EAT-lancet commission on healthy, sustainable, and just food systems. Lancet. (2025) 406:1625–700. doi: 10.1016/S0140-6736(25)01201-2 41046857

[6] Clark M Tilman D. Comparative analysis of environmental impacts of agricultural production systems, agricultural input efficiency, and food choice. Environ Res Lett. (2017) 12:064016. doi: 10.1088/1748-9326/aa6cd5

[7] Remans R Flynn DFB DeClerck F Diru W Fanzo J Gaynor K . Assessing nutritional diversity of cropping systems in African villages. PLoS One. (2011) 6:e21235. doi: 10.1371/journal.pone.0021235, 21698127 PMC3116903

[8] Herrero M Havlík P Valin H Notenbaert A Rufino MC Thornton PK . Biomass use, production, feed efficiencies, and greenhouse gas emissions from global livestock systems. Proc Natl Acad Sci USA. (2013) 110:20888–93. doi: 10.1073/pnas.1308149110, 24344273 PMC3876224

[9] Johnston JL Fanzo JC Cogill B. Understanding sustainable diets: a descriptive analysis of the determinants and processes that influence diets and their impact on health, food security, and environmental sustainability. Adv Nutr. (2014) 5:418–29. doi: 10.3945/an.113.005553, 25022991 PMC4085190

[10] FAO & WHO. Sustainable Healthy Diets - Guiding Principles. Rome: FAO & WHO (2019).

[11] Burlingame B Dernini S. Food and Agriculture Organization of the United Nations Bioversity International. Sustainable Diets and Biodiversity - Directions and Solutions for Policy Research and Action Proceedings of the International Scientific Symposium Biodiversity and Sustainable Diets United against Hunger.FAO (2012). Available at: https://cgspace.cgiar.org/items/244c6c6b-6bf0-4cf4-ab5b-4598716bd6e8

[12] Willett WC Sacks F Trichopoulou A Drescher G Ferro-Luzzi A Helsing E . Mediterranean diet pyramid: a cultural model for healthy eating. Am J Clin Nutr. (1995) 61:1402S–6S. doi: 10.1093/ajcn/61.6.1402S, 7754995

[13] Angelino D Bonaccio M Ruggiero E Formisano A Dello Russo M Lauria F . From global to local: comparing the EAT-lancet planetary health diets with the traditional Mediterranean diet across Mediterranean countries. Int J Food Sci Nutr. (2026) 77:314–25. doi: 10.1080/09637486.2026.2676223, 42206968

[14] Guasch-Ferré M Willett WC. The Mediterranean diet and health: a comprehensive overview. J Intern Med. (2021) 290:549–66. doi: 10.1111/joim.13333, 34423871

[15] Dernini S Berry EM Serra-Majem L La Vecchia C Capone R Medina FX . Med diet 4.0: the Mediterranean diet with four sustainable benefits. Public Health Nutr. (2017) 20:1322–30. doi: 10.1017/S1368980016003177, 28003037 PMC10261651

[16] Fresán U Martínez-Gonzalez M-A Sabaté J Bes-Rastrollo M. The Mediterranean diet, an environmentally friendly option: evidence from the Seguimiento Universidad de Navarra (SUN) cohort. Public Health Nutr. (2018) 21:1573–82. doi: 10.1017/S1368980017003986, 29380717 PMC10261578

[17] Dinu M Pagliai G Casini A Sofi F. Mediterranean diet and multiple health outcomes: an umbrella review of meta-analyses of observational studies and randomised trials. Eur J Clin Nutr. (2018) 72:30–43. doi: 10.1038/ejcn.2017.58, 28488692

[18] Dinu M Pagliai G Angelino D Rosi A Dall’Asta M Bresciani L . Effects of popular diets on anthropometric and cardiometabolic parameters: an umbrella review of meta-analyses of randomized controlled trials. Adv Nutr. (2020) 11:815–33. doi: 10.1093/advances/nmaa006 PMC7360456 32059053

[19] Jurado-Gonzalez P Xavier Medina F Bach-Faig A. Barriers to home food preparation and healthy eating among university students in Catalonia. Appetite. (2024) 194:107159. doi: 10.1016/j.appet.2023.107159, 38103793

[20] Satia-Abouta J Patterson RE Neuhouser ML Elder J. Dietary acculturation. J Am Diet Assoc. (2002) 102:1105–18. doi: 10.1016/S0002-8223(02)90247-6, 12171455

[21] Mensah FZ Lane KE Richardson LD. Determinants of eating behaviour in black, Asian and minority ethnic (BAME) university students when living at and away from home: with a focus on the influence of food enculturation and food acculturation. Appetite. (2022) 171:105932. doi: 10.1016/j.appet.2022.105932, 35051546

[22] Shi Y Lukomskyj N Allman-Farinelli M. Food access, dietary acculturation, and food insecurity among international tertiary education students: a scoping review. Nutrition. (2021) 85:111100. doi: 10.1016/j.nut.2020.111100, 33545541

[23] Almohanna A Conforti F Eigel W Barbeau W. Impact of dietary acculturation on the food habits, weight, blood pressure, and fasting blood glucose levels of international college students. J Am Coll Heal. (2015) 63:307–14. doi: 10.1080/07448481.2015.1025075, 25774756

[24] Hilal S Indrayathi PA Kolozsvári LR Torzsa P Rurik I. Psychosocial and academic implications of food insecurity among international students: a qualitative study in Hungary. Nutrients. (2025) 17:3300. doi: 10.3390/nu17203300, 41156552 PMC12567516

[25] Arrazat L Nicklaus S de Lauzon-Guillain B Marty L. Behavioural determinants of healthy and environmentally friendly diets in French university students. Appetite. (2024) 200:107532. doi: 10.1016/j.appet.2024.107532, 38815688

[26] Alakaam AA Castellanos DC Bodzio J Harrison L. The factors that influence dietary habits among international students in the United States. JIS. (2015) 5:104–20. doi: 10.32674/jis.v5i2.428

[27] Neri L d CL Fiorini S Bosio F Guglielmetti M Ferraro OE Tagliabue A . Nutritional health assistance for adapting food culture to the Mediterranean pattern for international students: study protocol. Int J Food Sci Nutr. (2025) 76:725–35. doi: 10.1080/09637486.2025.2561711, 40995823

[28] Marventano S Mistretta A Platania A Galvano F Grosso G. Reliability and relative validity of a food frequency questionnaire for Italian adults living in Sicily, southern Italy. Int J Food Sci Nutr. (2016) 67:857–64. doi: 10.1080/09637486.2016.1198893, 27333325

[29] Sofi F Dinu M Pagliai G Marcucci R Casini A. Validation of a literature-based adherence score to Mediterranean diet: the MEDI-LITE score. Int J Food Sci Nutr. (2017) 68:757–62. doi: 10.1080/09637486.2017.1287884, 28276908

[30] CREA. Banca Dati di Composizione degli Alimenti per Studi Epidemiologici in Italia (BDA. Available online at: http://www.bda-ieo.it (Accessed September 5, 2025)

[31] Aureli V Vici G Giosuè A Devecchi A Martini D. One diet, many indices: heterogeneity in measuring adherence to the EAT-lancet planetary health diet. Int J Food Sci Nutr. (2026) 77:426–37. doi: 10.1080/09637486.2026.2694533, 42405417

[32] Cacau LT De Carli E de Carvalho AM Lotufo PA Moreno LA Bensenor IM . Development and validation of an index based on EAT-lancet recommendations: the planetary health diet index. Nutrients. (2021) 13:1698. doi: 10.3390/nu13051698, 34067774 PMC8156093

[33] Cohen J. Statistical power analysis. Curr Dir Psychol Sci. (1992) 1:98–101. doi: 10.1111/1467-8721.ep10768783

[34] Psicometria Online Academy. *Calculadora de Análise de Poder Estatístico*. (2026) Available online at: https://psicometriaonline.com.br/ (Accessed August 12, 2026)

[35] Cohen J. Statistical Power Analysis for the Behavioral Sciences. 2nd ed. New York: Lawrence Erlbaum Associates (1988).

[36] JASP Team. JASP. Amsterdam, The Netherlands: JSP Team (2024).

[37] Alakaam A Willyard A. Eating habits and dietary acculturation effects among international college students in the United States. AMS Public Health. (2020) 7:228–40. doi: 10.3934/publichealth.2020020, 32617352 PMC7327396

[38] Shi Y Hayba N Allman-Farinelli M. International tertiary education students experienced difficulties in dietary transitions in Australia: a qualitative study. Health Promot J Austr. (2024) 35:165–75. doi: 10.1002/hpja.728, 37029086

[39] Mahmoodi M George RM Gokhale D. Dietary acculturation of international students in Pune, India: a cross-sectional study. Nutr Health. (2022) 28:271–6. doi: 10.1177/02601060211019678, 34187230

[40] Kremmyda L-S Papadaki A Hondros G Kapsokefalou M Scott JA. Differentiating between the effect of rapid dietary acculturation and the effect of living away from home for the first time, on the diets of Greek students studying in Glasgow. Appetite. (2008) 50:455–63. doi: 10.1016/j.appet.2007.09.014, 17997195

[41] Papadaki A Hondros G Scott J Kapsokefalou M. Eating habits of university students living at, or away from home in Greece. Appetite. (2007) 49:169–76. doi: 10.1016/j.appet.2007.01.008 17368642

[42] Béné C Oosterveer P Lamotte L Brouwer ID de Haan S Prager SD . When food systems meet sustainability – current narratives and implications for actions. World Dev. (2019) 113:116–30. doi: 10.1016/j.worlddev.2018.08.011

[43] Bernardo GL Jomori MM Fernandes AC Colussi CF Condrasky MD Proença RP d C. Nutrition and culinary in the kitchen program: a randomized controlled intervention to promote cooking skills and healthy eating in university students - study protocol. Nutr J. (2017) 16:83. doi: 10.1186/s12937-017-0305-y, 29262811 PMC5738807

[44] Dezanetti T Quinaud RT Caraher M Jomori MM. Meal preparation and consumption before and during the COVID-19 pandemic: the relationship with cooking skills of Brazilian university students. Appetite. (2022) 175:106036. doi: 10.1016/j.appet.2022.106036, 35429579 PMC9007752

[45] Watanabe JA Nieto JA Suarez-Diéguez T Silva M. Influence of culinary skills on ultraprocessed food consumption and Mediterranean diet adherence: an integrative review. Nutrition. (2024) 121:112354. doi: 10.1016/j.nut.2024.112354, 38458144

[46] Neuenschwander M Stadelmaier J Eble J Grummich K Szczerba E Kiesswetter E . Substitution of animal-based with plant-based foods on cardiometabolic health and all-cause mortality: a systematic review and meta-analysis of prospective studies. BMC Med. (2023) 21:404. doi: 10.1186/s12916-023-03093-1, 37968628 PMC10652524

[47] Quarta S Siculella L Levante A Carluccio MA Calabriso N Scoditti E . Association between Mediterranean lifestyle and perception of well-being and distress in a sample population of university Italian students. Int J Food Sci Nutr. (2023) 74:556–67. doi: 10.1080/09637486.2023.2232129, 37431100

[48] Moullan Y Jusot F. Why is the “healthy immigrant effect” different between European countries? Eur J Pub Health. (2014) 24:80–6. doi: 10.1093/eurpub/cku112, 25108002

[49] Tirelli C Pilar Martínez-Ruiz M Gómez-Ladrón-De-Guevara R. Major influences on buying decision processes by international university students. Differences by continent of origin. Appetite. (2013) 71:104–12. doi: 10.1016/j.appet.2013.08.002 23941704

[50] Spruance LA Vo T. Race/ethnicity predicts school meal participation. J Sch Health. (2023) 93:305–12. doi: 10.1111/josh.13270, 36377073

[51] Bucciarelli V Moscucci F Cocchi C Nodari S Sciomer S Gallina S . Climate change versus Mediterranean diet: a hazardous struggle for the women’s heart. Am Heart J Plus. (2024) 45:100431. doi: 10.1016/j.ahjo.2024.100431, 39175598 PMC11340622

