# Supplementary material for: Promoting alignment with Mediterranean and planetary health diet principles among international students: an exploratory pilot study of the MeditEat project
Source: Front Nutr. 2026 Oct 21;13:1946581. doi: 10.3389/fnut.2026.1946581 (PMC13762446; doi:10.3389/fnut.2026.1946581)
Supplement: Supplementary file 1 [file Supplementary_file_1.docx]

Supplementary Material

| Table S1 - Planetary Health index components, standards for scoring (caloric densities), and corresponding point values. The Food group Animal Fats was excluded from the original version of this index. | | | | | | |
| --- | --- | --- | --- | --- | --- | --- |
| Component Category | Food Group / Metric | 0 Points | 5 Points (Intermediate Range) | 10 Points (Target) | 5 Points (Intermediate Range) | 0 Points |
| Adequacy | Nuts and peanuts | 0.0 | 0.1 < value < 11.6 | ≥ 11.6 | — | — |
|  | Legumes | 0.0 | 0.1 < value < 11.3 | ≥ 11.3 | — | — |
|  | Fruits | 0.0 | 0.1 < value < 5.0 | ≥ 5.0 | — | — |
|  | Vegetables | 0.0 | 0.1 < value < 3.1 | ≥ 3.1 | — | — |
|  | Whole cereals | 0.0 | 0.1 < value < 32.4 | ≥ 32.4 | — | — |
| Optimum | Eggs | 0.0 | 0.1 < value < 0.8 | 0.8 | 0.8 < value < 1.5 | ≥ 1.5 |
|  | Fish and seafood | 0.0 | 0.1 < value < 1.6 | 1.6 | 1.6 < value < 5.7 | ≥ 5.7 |
|  | Tubers and potatoes | 0.0 | 0.1 < value < 1.6 | 1.6 | 1.6 < value < 3.1 | ≥ 3.1 |
|  | Dairy | 0.0 | 0.1 < value < 6. | 6.1 | 6.1 < value < 12.2 | ≥ 12.2 |
|  | Vegetable oils | 0.0 | 0.1 < value < 16.5 | 16.5 | 16.5 < value < 30.7 | ≥ 30.7 |
| Ratio | Dark green vegetable / total ratio | 0.0 | 0.1 < value < 29.5 | 29.5 | 29.5 < value < 100 | 100 |
|  | Red vegetable / total ratio | 0.0 | 0.1 < value < 38.5 | 38.5 | 38.5 < value < 100 | 100 |
| Moderation | Red meat | ≥ 2.4 | 0.1 < value < 2.4 | 0.0 | — | — |
|  | Chicken and substitutes | ≥ 5.0 | 0.1< value < 5.0 | 0.0 | — | — |
|  | Added sugars | ≥ 4.8 | 0.1 < value < 4.8 | 0.0 | — | — |

Figure S1: Diagram of participant recruitment, selection, and retention.

Broad Recruitment Outreach

(University-wide emails via International Office & Student Fairs)

Screened for Eligibility 🡪 Excluded: 0

(n = 17)

Enrolled / Baseline

(n = 17)

Intervention & Follow-up 🡪 Discontinued (n = 2):

- Reasons:
  - Time constraints / workload (n = 1)
  - Mental health / depression (n = 1)

Final Analysis Sample

(n = 15)

(Complete-case analysis at T0, T3, T6)

**Figure S2: Longitudinal changes in Planetary Health Diet Index (PHDI) total score across intervention time points (T0, T3, T6).**

a	b	b

^a,b^ Different letters indicate statistically significant post-hoc pairwise differences (p < 0.05, Holm correction).

**Figure S3: Longitudinal changes in Planetary Health Diet Index (PHDI) Nuts score across intervention time points (T0, T3, T6).**

**Figure S4: Longitudinal changes in Planetary Health Diet Index (PHDI) Added sugars score across intervention time points (T0, T3, T6).**

**Figure S5: Longitudinal changes in Planetary Health Diet Index (PHDI) cereals score across intervention time points (T0, T3, T6).**

**Figure S6: Longitudinal changes in Planetary Health Diet Index (PHDI) Chicken and subs score across intervention time points (T0, T3, T6).**

**Figure S7: Longitudinal changes in Planetary Health Diet Index (PHDI) Dairy score across intervention time points (T0, T3, T6).**

**Figure S8: Longitudinal changes in Planetary Health Diet Index (PHDI) Eggs score across intervention time points (T0, T3, T6).**

**Figure S9: Longitudinal changes in Planetary Health Diet Index (PHDI) Fish and Seafood score across intervention time points (T0, T3, T6).**

**Figure S10: Longitudinal changes in Planetary Health Diet Index (PHDI) Fruits score across intervention time points (T0, T3, T6).**

**Figure S11: Longitudinal changes in Planetary Health Diet Index (PHDI) Legumes score across intervention time points (T0, T3, T6).**

**Figure S12: Longitudinal changes in Planetary Health Diet Index (PHDI) Red Meat score across intervention time points (T0, T3, T6).**

**Figure S13: Longitudinal changes in Planetary Health Diet Index (PHDI) Tubers and potatoes score across intervention time points (T0, T3, T6).**

**Figure S14: Longitudinal changes in Planetary Health Diet Index (PHDI) vegetable oils score across intervention time points (T0, T3, T6).**

a	b	b

^a,b^ Different letters indicate statistically significant post-hoc pairwise differences (p < 0.05, Holm correction).

**Figure S15: Longitudinal changes in Planetary Health Diet Index (PHDI) Vegetables score across intervention time points (T0, T3, T6).**
